# Haplotype Distribution and Evolutionary Pattern of miR-17 and miR-124 Families Based on Population Analysis

**DOI:** 10.1371/journal.pone.0007944

**Published:** 2009-11-23

**Authors:** Li Guo, Beili Sun, Fei Sang, Wei Wang, Zuhong Lu

**Affiliations:** 1 State Key Laboratory of Bioelectronics, School of Biological Science and Medical Engineering, Southeast University, Nanjing, China; 2 Key Laboratory of Child Development and Learning Science of Ministry of Education, Southeast University, Nanjing, China; University of California San Diego, United States of America

## Abstract

**Background:**

MicroRNAs (miRNAs) are small, endogenously expressed non-coding RNAs that regulate mRNAs post-transcriptionally. Previous studies have explored miRNA evolutionary trend, but evolutionary history and pattern in the miRNA world are still not fully clear. In the paper, we intended to analyze miRNA haplotype distribution and evolutionary network by analyzing miRNA sequences of miR-17 and miR-124 families across animal species as special populations.

**Principal Findings:**

31 haplotypes were detected in miR-17 family while only 9 haplotypes were defined in miR-124 family. The complex miR-17 family was mainly distributed in vertebrates, but miR-124 was shared by more animal species from *Caenorhabditis* to *Homo* and had a wide distribution spectrum. Some haplotypes of the two miRNA families appeared discontinuous distributions across animals. Compared with a simple phylogenetic network in miR-124 family, miR-17 family indicated a complex network with some median vectors that might be lost ancestral or potential miRNA haplotypes. By analyzing different miRNAs across 12 animal species, we found these small RNAs showed different haplotype diversities, haplotype distributions and phylogenetic networks.

**Conclusions:**

Different miRNAs had quite different haplotype distributions and evolutionary patterns. Discontinuous distributions of miRNAs and median vectors in phylogenetic networks implied more members in the miRNA world. miRNA may be an excellent phylogenetic marker to discover its evolutionary history and pattern across the animal kingdom.

## Introduction

MicroRNAs (miRNAs), a newly discovered class of non-protein-coding small RNAs, play pivotal roles in negatively regulating gene expression by targeting mRNAs for translational repression, cleavage or destabilization post-transcriptionally in animals and plants [1], [2], [3], [4], [5]. It is well known that the small RNAs are very well conserved phylogenetically across large evolutionary distances like vertebrates and fruit flies [6], [7], [8], and some have similar developmental roles in diverse species [9]. Many miRNAs are expressed in particular developmental contexts at specific times, suggesting miRNAs could have played a fundamental evolutionary role in increasing phenotypic diversity and complexity with the number of miRNAs [10], [11], [12], [13]. Because of these features, the small non-coding RNA has captured the attention of investigators who are interested in its evolution and a broad phylogenetic perspective. Indeed, previous studies show miRNA evolutionary history can be accurately reconstructed to elucidate evolutionary history and trend across the animal kingdom because miRNAs are strongly conserved in primary sequence and rarely secondarily lost [10], [14], [15], [16]. Compared with other components of gene regulatory networks, largely conserved miRNA molecules show a different evolutionary pattern with a very compelling feature [9], [17]. Mature miRNA sequences and their precursor sizes differ greatly between poriferans, cnidarians, and bilaterians, which suggests relatively dynamic evolution [12]. Despite well evolutionarily conserved, mature miRNAs show evolutionary stable “seed shifting” among animal species [17]. miRNA repertoire can be reshaped by loss/derivation events, which suggests the profound reorganization of the miRNA repertoire integral to evolution [18]. Increasing evidences indicate that many miRNAs appear in clusters on a single polycistronic transcript, and the evolutionary history of clusters may be governed by duplication and subsequent loss of individual miRNAs from the resulting paralogous clusters [19], [20].

Although a few more studies have explored miRNA evolutionary information across some animal species, little is known about haplotype distribution and evolutionary pattern and trend for miRNA by analyzing miRNA sequences as the whole population across the animal kingdom. According to a set of distinctive features, miRNAs can be analyzed as a special population across animal species. Firstly, accumulating evidences show that miRNAs appear to shape gene expression since at least very early time in animal evolution [12], suggesting that the small non-coding RNAs are ancient RNAs. Secondly, miRNAs are highly evolutionarily conserved across large evolutionary distances in animals from worms to humans [21], [22], which indicates that the small RNAs are perfectly conserved across the animal kingdom. Hence, it is a powerful strategy to analyze miRNAs as a special population across the animal kingdom to study haplotype distribution and evolutionary network to infer potential evolutionary history, pattern and trend in the miRNA world.

Here, to dissect miRNA haplotype diversity and distribution, evolutionary pattern across the animal kingdom, miR-17 and miR-124 families were analyzed as special populations. The two objects of study are typically representative miRNA families in the miRNA world. A complex family based on precursor sequences, miR-17 family, consists of five homologous members: miR-17, miR-18, miR-20, miR-93, and miR-106. miR-124 family is a single gene and has no homologous members. Moreover, because all of these miRNAs (miR-17, miR-18, miR-20, miR-93, miR-106, and miR-124) are detected in 12 animal species, further analysis of the evolutionary pattern for the different miRNAs across the same kinds of animals can be performed. Every animal species was studied as a special individual in the miRNA population, and simultaneously different precursor sequences for the same mature miRNA sequence were considered. All the complete mature miRNA sequences were analyzed to define haplotypes. Combining with the available data, we attempted to provide some analysis of the haplotype diversity and distribution, and evolutionary network of miRNA families by cross-species comparison. With an emphasis on evolutionary question, potential evolutionary trend and connection between functional and evolutionary diversities were discussed. In order to dissect diversity of miRNA evolutionary pattern in the same kinds of animal species, the six kinds of miRNAs across 12 animal species (miR-17, miR-18, miR-20, miR-93, miR-106, and miR-124) were further analyzed. By comparison and analysis, the plausible evolutionary history and trend, potential relationship between functional and evolutionary diversity of miR-124 and miR-17 gene families were discussed, especially for the evolutionary pattern between different miRNAs across the same kinds of animal species.

## Results

### miR-17 Family

**Figure 1:**
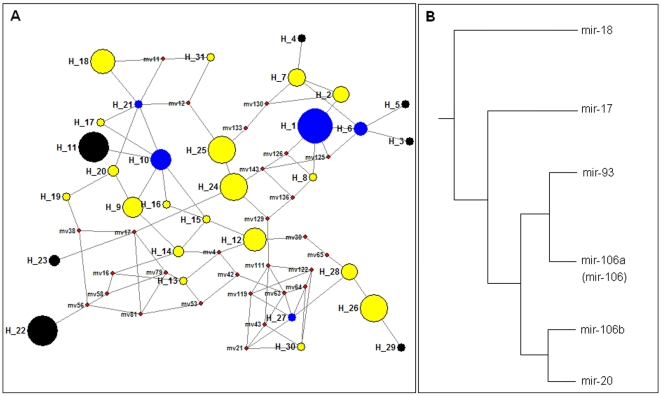
Phylogenetic tree of precursors (A) and network of miR-17 family (B). (**A**) Simplified Neighbor-joining tree of precursors showing relationships between the five members in miR-17 family. (**B**) Phylogenetic network showing evolutionary relationships between haplotypes in miR-17 family. In the network, the size of the circle indicates relative frequency of the haplotype. The blue circles indicate the most ancestral haplotypes; the black circles indicate the most recent haplotypes; the red circles are median vectors (hypothesized sequences); other haplotypes are characterized yellow circles.

A total of 144 mature miRNA sequences (different precursor sequences for the same mature miRNA sequence were also considered), from 24 animal species, were identified 31 haplotypes (Hd = 0.94, π = 0.17) with gaps or missing sites considered (Table S1). Phylogenetic network of miR-17 family based on maximum parsimony option indicated complex structure with many median vectors (red circles) that were lost ancestral or potential mature miRNA sequences (Figure 1A). H_1 was the most dominant haplotype and shared by 18 animals (Table S1, Figure 1A). Some haplotypes located at the central positions by star-like phylogenies at least 5 clades were the most ancestral haplotypes, such as H_1, H_6, H_10, H_21, and H_27 (Figure 1A, blue circles). Phylogenetic clades of 7 haplotypes (H_3, H_4, H_5, H_11, H_22, H_23, and H_29), derived directly or indirectly from the network torso structure and ancient haplotypes or lost potential haplotypes, and these haplotypes were the most recent miRNAs (Figure 1A, black circles). Phylogenetic distribution based on precursor sequences of miR-17 family indicated most of the members were clustered single groups, and the outer cluster was mir-18 (Figure 1B), but almost every monophyletic group showed a private and complex evolutionary pattern (tree not shown).

**Figure 2:**
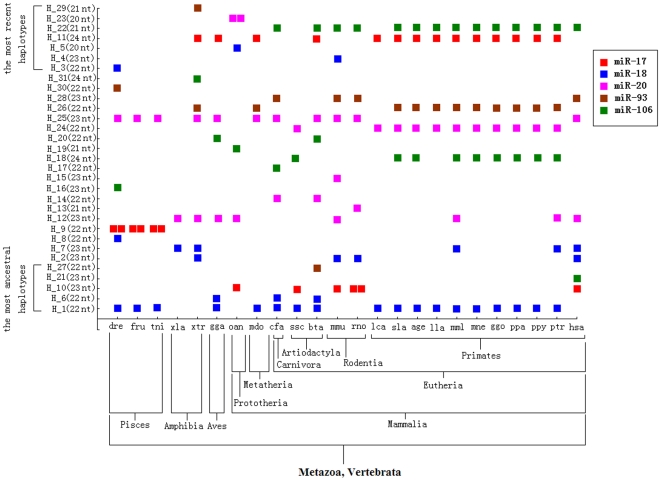
The haplotype distribution of miR-17 family and evolutionary position of 24 animal species. Continuous symbols indicate the miRNA sequence can be produced by two precursors. For abbreviations, see Table 1.

So far, haplotype distribution across the animal kingdom revealed that the miR-17 family was detected from *Danio rerio* to *Homo sapiens* (Figure 2). Many miRNA haplotypes were rare or private haplotypes that were only shared by 1∼2 animal species, and others showed discontinuous distribution models across animal species, such as H_10, H_12, H_25, and H_26 (Figure 2). For example, H_10 was shared by *Ornithorhynchus anatinus*, *Sus scrofa*, *Mus musculus*, *Rattus norvegicus*, and *Homo sapiens*, despite of a larger evolutionary distance between *Ornithorhynchus anatinus* and *Homo sapiens*, and most of primates were not detected of the miRNA haplotype (Figure 2). Similarly, some members of miR-17 family, such as miR-93 and miR-106, showed discontinuous distribution across the 24 animals because some specific animals were not detected these miRNA haplotypes (Figure 2). The most ancestral haplotypes derived many recent haplotypes, but many of them had lost dominant positions except for H_1, which still was the most dominant haplotype and shared by many animal species (Figure 2). The most recent haplotypes, H_11 and H_22, were selected as common haplotypes by many mammalian species. Some evolutionary intermediates were also shared as dominant miRNA haplotypes in metazoans (Figure 2). Generally, the members of the gene family located on 2∼4 chromosomes, and these genes on the same chromosome always appeared in a cluster according to the distance between genes <10 kb and shared the same core promoter to be a single transcript.

### miR-124 Family

**Figure 3:**
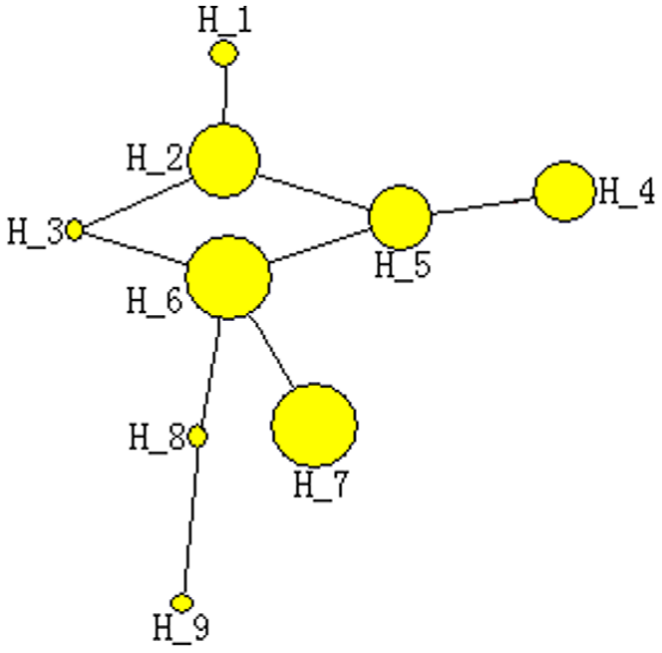
Network showing phylogenetic relationships between haplotypes in miR-124 family.

**Figure 4:**
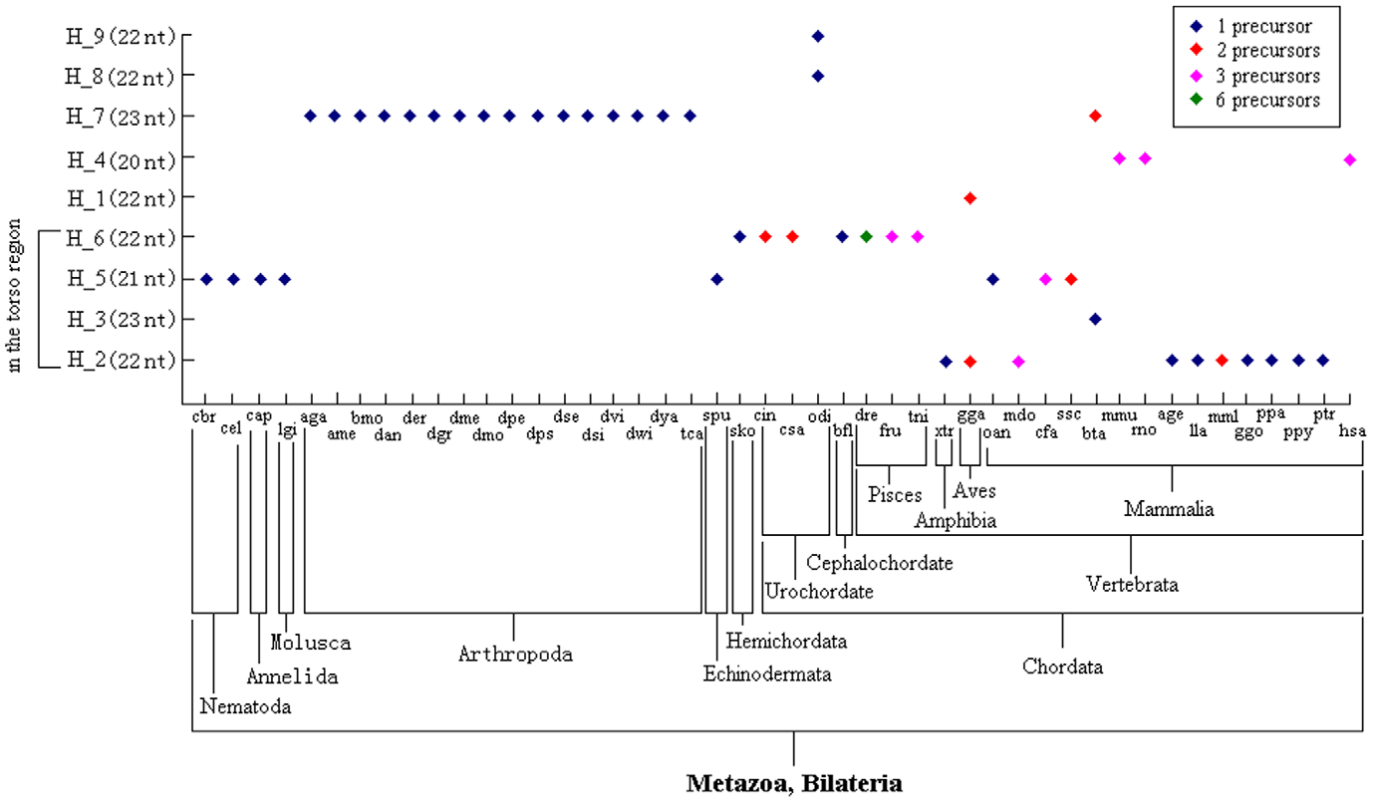
The haplotype distribution of miR-124 family and evolutionary position of 46 animal species. The four haplotypes (H_2, H_3, H_5, and H_6) were located in the torso region of network. For abbreviations, see Table 1.

Compared with the complex miR-17 family, miR-124 family was only identified 9 haplotypes (Hd = 0.83, π = 0.01) based on 75 mature miRNA sequences considering gaps or missing sites (Table S2). The phylogenetic relationships between the 9 haplotypes showed a simple network without lost or potential haplotypes (median vectors) detected (Figure 3). Some haplotypes in the central positions and torso structure (H_2, H_3, H_5, and H_6) were basal and ancient haplotypes than other haplotypes (Figure 3). H_2 and H_7 were selected as the most dominant haplotypes with higher rates of appearance (Table S2, Figure 3, Figure 4).

The miR-124 family was detected in 46 animal species from *Caenorhabditis* to *Homo sapiens* (Figure 4). Four haplotypes were private haplotypes, and others appeared discontinuous distributions across phylogenetic taxa, especially if the boundary was *Strongylocentrotus purpuratus* (Figure 4). The ancestral haplotypes were widely distributed in many animal species, especially in most of chordates (only several chordate animals shared recent haplotypes, for example, *Oikopleura dioica*, *Mus musculus*, *Rattus norvegicus*, and *Homo sapiens*. *Gallus gallus* and *Bos taurus* were detected ancestral and recent haplotypes). Although these ancestral haplotypes had derived directly or indirectly many recent haplotypes, many of them still were dominant haplotypes and shared by many animal species (Figure 4). Lower animals, such as nematodes, annelids, molluscs, arthropods, echinodermates, and hemichordates, were found only one miRNA haplotype (H_5, H_6, or H_7) that was produced by one precursor. Interestingly, H_5, H_6, and H_7 were also selected by some chordates, while many of them were detected 2∼6 precursors to be processed the same mature miRNA sequence (Figure 4). Some of these precursors could locate on the same chromosome (for example, hsa-mir-124-1 and hsa-mir-124-2 located on chromosome 8 in *Homo sapiens*, mdo-mir-124a-1 and mdo-mir-124a-3 located on chromosome 1 in *Monodelphis domestica*, mml-mir-124a-1 and mml-mir-124a-2 located on chromosome 8 in *Macaca mulatta*) but in the distant range (>10,000 kb). No data and evidence indicated that the gene was composed of cluster with other genes.

### Six Kinds of miRNAs in 12 Animal Species

**Figure 5:**
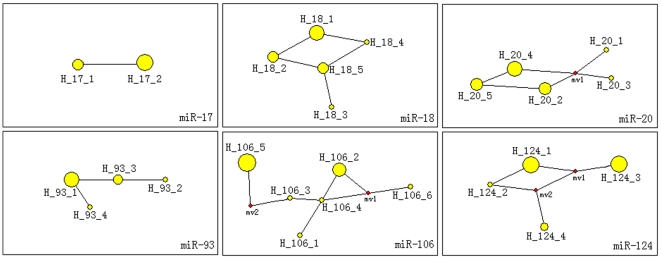
Networks of different miRNAs across 12 animal species showing corresponding phylogenetic relationships between haplotypes. For abbreviations, see Table 1.

To infer potential evolutionary pattern across miRNAs in the same kinds of animal species, all members of miR-17 family (miR-17, miR-18, miR-20, miR-93, and miR-106) and miR-124 mature sequences were analyzed across 12 animals. The amount of variable sites were flexible (1∼7) for different miRNAs despite all of them were detected in 12 animal species, and haplotypes also showed evident diversity (Table 1). The haplotype distributions showed that *Mus musculus, Rattus norvegicus*, and *Homo sapiens* always shared the common haplotypes (H_17_1, H_18_2, H_20_5, H_93_3, H_106_5, and H_124_3) despite many other primates were analyzed simultaneously (Table 1). Phylogenetic networks revealed different evolutionary patterns for the different miRNAs, but none of these showed complex networks (Figure 5). Only 1∼2 median vectors were found in networks of miR-20, miR-106, and miR-124. Interestingly, no median vectors were found in network of miR-124 family based on 9 haplotypes across 46 animal species (Figure 3).

**Table 1:** Haplotype distributions for six kinds of miRNAs across 12 animal species.

Haplotype (frequency)	xtr	bta	mmu	rno	age	lla	mml	ggo	ppa	ppy	ptr	hsa
H_17_1 (4)			1	2								1
H_17_2 (9)	1	1			1	1	1	1	1	1	1	
H_18_1 (8)		1			1	1	1	1	1	1	1	
H_18_2 (4)	1		1	1								1
H_18_3 (1)			1									
H_18_4 (1)		1										
H_18_5 (4)	1						1				1	1
H_20_1 (1)				1								
H_20_2 (5)	1		1				1				1	1
H_20_3 (1)		1										
H_20_4 (7)					1	1	1	1	1	1	1	
H_20_5 (5)	1	1	1	1								1
H_93_1 (8)	1				1	1	1	1	1	1	1	
H_93_2 (1)		1										
H_93_3 (3)			1	1								1
H_93_4 (1)	1											
H_106_1 (1)			1									
H_106_2 (6)					1		1	1	1	1	1	
H_106_3 (1)		1										
H_106_4 (1)												1
H_106_5 (11)		1	1	1	1	1	1	1	1	1	1	1
H_106_6 (1)	1											
H_124_1 (9)	1				1	1	2	1	1	1	1	
H_124_2 (1)		1										
H_124_3 (9)			3	3								3
H_124_4 (2)		2										
Total	9	11	11	10	7	6	10	7	7	7	9	11

The number (1, 2, 3) shows that miRNA sequence can be produced by different precursors.

Taxonomic abbreviations: aga, *Anopheles gambiae*; age, *Ateles geoffroyi*; ame, *Apis mellifera*; bfl, *Branchiostoma floridae*; bmo, *Bombyx mori*; bta, *Bos taurus*; cap, *Capitella sp.l*; cbr, *Caenorhabditis elegans*; cel, *Caenorhabditis elegans*; cfa, *Canis familiaris*; cin, *Ciona intestinalis*; csa, *Ciona savignyi*; dan, *Drosophila ananassae*; der, *Drosophila erecta*; dgr, *Drosophila grimshawi*; dme, *Drosophila melanogaster*; dmo, *Drosophila mojavensis*; dpe, *Drosophila persimilis*; dps, *Drosophila pseudoobscura*; dre, *Danio rerio*; dse, *Drosophila sechellia*; dsi, *Drosophila simulans*; dvi, *Drosophila virilis*; dwi, *Drosophila willistoni*; dya, *Drosophila yakuba*; fru, *Fugu rubripes*; gga, *Gallus gallus*; ggo, *Gorilla gorilla*; hsa, *Homo sapiens*; lca, *Lemur catta*; lgi, *Lottia gigantea*; lla, *Lagothrix lagotricha*; mdo, *Monodelphis domestica*; mml, *Macaca mulatta*; mmu, *Mus musculus*; mne, *Macaca nemestrina*; oan, *Ornithorhynchus anatinus*; odi, *Oikopleura dioica*; ppa, *Pan paniscus*; ppy, *Pongo pygmaeus*; ptr, *Pan troglodytes*; rno, *Rattus norvegicus*; sko, *Saccoglossus kowalevskii*; sla, *Saguinus labiatus*; spu, *Strongylocentrotus purpuratus*; ssc, *Sus scrofa*; tca, *Tribolium castaneum*; tni, *Tetraodon nigroviridis*; xla, *Xenopus laevis*; xtr, *Xenopus tropi*.

## Discussion

### Haplotype Distribution

Despite of well evolutionary conserved across the animal kingdom in the miRNA world, it was evident that the great divergence of haplotype distributions between miR-17 and miR-124 families. The miR-17 family was detected in 24 vertebrate animals, while miR-124 was found in 46 animal species (Figure 2, Figure 4). Compared with miR-17 family, miR-124 had a wide distribution spectrum from *Caenorhabditis* to *Homo* (Figure 2, Figure 4). Even though different animal species were identified with a larger evolutionary distance, miR-124 still shared the same miRNA haplotype (such as H_5 and H_7) and showed highly evolutionary conservation from *Caenorhabditis* to *Homo* (Figure 4). Nevertheless, the hierarchical distribution was evident across the 46 animal species, especially if the boundary was *Strongylocentrotus purpuratus* (Figure 4). The miRNA can be yielded from 2∼6 precursor sequences in some chordate animals, while most of miRNA sequences in miR-17 family were produced by one precursor (Figure 2, Figure 4). We surmised the haplotype distributions of the two miRNAs maybe reveal evolutionary history and trend in individual and the animal kingdom. For example, the complex miR-17 family was not detected in some lower animals (such as *Caenorhabditis* and *Capitella*) but only found in some vertebrates, which revealed the miRNA might be lost selection advantage and gotten rid of in some lower bilaterian animals or derived other miRNA gene with loss of original gene during evolutionary fluctuation history. However, miR-124 was still stable selected with well conserved in sequence by some lower animals such as *Caenorhabditis* and *Capitella*. The highly conserved miRNA may experience critical and severe selection during evolutionary fluctuation with multiple and complex gene expansion and frequent mutation, but important functional need ensured great selection advantage through the whole complex evolutionary history. Therefore, although less miRNA haplotypes were detected across the 46 animals, miR-124 of chordates can be produced by 2∼6 precursors (Figure 4). The multiple precursors might be a response to complex history of duplication with less or no variation in miRNA sequences. All of these results indicated miR-124 was a quite important and essential miRNA that played the same critical function of pivotal biological processes from *Caenorhabditis* to *Homo*. In fact, some recent studies show that miR-124 is one of the most abundantly expressed miRNAs in the nervous system and contributes to the development of nervous system, being widely expressed in neurons in the brain, retina and spinal cord [23], [24], [25]. In *Drosophila*, the miRNA is expressed exclusively in neuronal cells as they begin to differentiate [26], indicating that miR-124 may contribute to maintaining neuronal identity by suppressing nonneuronal genes in neurons [27]. Therefore, because of the critical function in nervous system, as a quite important miRNA, miR-124 contributes to different animals and highly conserved across animal kingdom.

Half of haplotypes of miR-17 and miR-124 families were rare or private haplotypes and only shared by 1∼2 animals, and others showed discontinuous distribution models across animal species (Figure 2, Figure 4). Rare or private haplotypes, could be ancestral miRNAs (such as H_21 and H_27 in miR-17 family) or recent miRNAs (such as H_3 and H_4 in miR-17 family) (Figure 2, Figure 4). If some ancestral haplotypes lost dominant position during evolutionary history, they would be selected by fewer animals or derived new miRNA sequences with loss of original miRNAs by editing the terminus regions especially the 3′ terminus regions. Although the terminus regions were modified, the identities (nucleotides 2∼8) always were not changed but “seed shifting” could be detected in some novel miRNA sequences (Table S1, Table S2). In fact, mature miRNAs always showed evolutionary stable “seed shifting” among different animal species [17]. Because of well evolutionarily conserved in the miRNA world, many of miRNA sequences were shared by more animals but showed discontinuous distributions across animal species (Figure 2, Figure 4). For example, H_1, from the member miR-18 in miR-17 family, was widely selected by 18 animal species, but some animals (*Xenopus laevis*, *Xenopus tropi*, *Ornithorhynchus anatinus*, *Mus musculus*, *Rattus norvegicus*, and *Homo sapiens*) were not detected the dominant haplotype although all of these animals were detected other intermediate or recent miR-18 haplotypes (Figure 2). Discontinuous distribution across the animal kingdom showed diversity of miRNA sequences based on certain seed sequence, and the sufficient variations flank mature miRNAs could contribute to the evolutionary diversification of these key regulatory genes [28]. The diversity of miRNA sequences in different animals might have close relationship with species diversity (as discussed below).

### Diversity of Evolutionary Pattern

Phylogenetic network of miR-17 family based on 31 haplotypes from *Danio rerio* to *Homo sapiens* revealed a complex pattern with many median vectors (red circles), which might be lost ancestral or potential mature miRNA sequences (Figure 1A). Recent haplotypes were evolved directly or indirectly from the ancestral haplotypes, but many of these origin miRNAs had lost dominant position or selective advantage in animal species except for H_1 (Table S1, Figure 1A, Figure 2). Compared with complex miR-17 family, network of miR-124 family based on 9 haplotypes showed a simple evolutionary pattern without median vectors (Figure 3). Most of ancient haplotypes still remained dominant positions despite they had derived directly or indirectly many recent miRNA sequences, while only one recent haplotype (H_7) was widely selected by many animal species (Table S2, Figure 3, Figure 4). The divergence between networks of the two miRNA families might imply different evolutionary patterns and rates. The complex evolutionary network of miR-17 family showed complex evolutionary history with a fast evolutionary rate, involved loss/derivation for original gene. miR-124 family indicated a very well conserved phylogenetically evolutionary process across the animal kingdom, and only 9 haplotypes were defined although 46 animal species were involved (Table S2, Figure 3, Figure 4). In fact, miR-17 gene family consists of five homologous members that locate on 2∼4 chromosomes. These members on the same chromosome always compose a cluster with other miRNAs (such as miR-19 and miR-92) according to the distance between genes <10 kb and share the same core promoter to be a single transcript. The precursors of miR-17 family, might be experienced through a complex history of duplication and loss of individual members as well as duplication of entire paralogs [20]. Therefore, complex evolutionary history involved loss/derivation for original genes and mature miRNAs showed today's evolutionary pattern (Figure 1). However, miR-124 family, a single gene family because no report indicated the miRNA had homologous genes and appeared in a miRNA cluster, showed a very well conserved phylogenetically evolutionary process across the animal kingdom.

Furthermore, to dissect diversity of evolutionary pattern in the miRNA world across the same kinds of animal species, miR-124 and the five members of the miR-17 gene family (miR-17, miR-18, miR-20, miR-93, and miR-106) – all of them were detected in 12 different animal species – were further analyzed. As shown in Table 1 and Figure 5, these miRNAs showed different haplotype diversities, dominant haplotypes, the number of median vectors, phylogenetic networks, and evolutionary patterns. No similar haplotype distributions of the six kinds of miRNAs were revealed despite all the miRNAs were identified in the same animal species (Table 1, Figure 5). Many haplotypes were defined common haplotypes because they were shared by at least two animal species in the study. Simple networks indicated conservative evolutionary patterns across the 12 specific animals, but divergence of networks revealed diversity between evolutionary patterns (Figure 5). Generally, no discussion of the evolution of gene regulation is complete without a consideration of the developmental roles of the regulators themselves, because these roles imply certain constraints on the evolvability of the regulatory relationships [29], [30], [31]. Therefore, although the small RNAs were well conserved across the animal kingdom, it was evident that the divergence of evolutionary process in different miRNAs and animal species (Table 1, Figure 5). All these findings suggested a positive relationship between evolutionary diversity and species diversity, and pointed to selective advantage during the evolutionary process. Finally, miRNAs are considered one of the largest gene families [2], [32], [33] and are dictated by individual as well as interactive effects of multiple evolutionary forces from genes and environments during evolutionary fluctuation history, such as mutation, recombination, duplication, and random genetic drift. Of course, any evolutionary force and process should not isolate with functional pressure despite which may be the final evolutionary aim or trend. How to detect more information for evolutionary history and pattern would be an important factor to recognize the interesting miRNA world.

### More Members in the miRNA World

As there are only 106 species in miRBase database (version 13.0, http://microrna.sanger.ac.uk/sequences), more miRNAs are still remain unknown especially for miRNAs that always are expressed in particular developmental contexts at specific times and in specific tissues [10], [11], [12], [13]. In the study, we also provided explicit proofs to convince more members in the miRNA world. Firstly, some members of miR-17 family, such as miR-93 and miR-106, showed discontinuous distribution across limited animal species (Figure 2). The result revealed that the miRNAs were not detected in some specific animals by experimental approach. However, these miRNAs might be expressed in specific tissues and at specific times. Limited studies based on traditional experimental approaches might neglect many miRNAs, especially for some miRNAs that are expressed at low levels. Secondly, some networks based on the complete miRNA sequences indicated many median vectors that might be lost ancestral or potential miRNA sequences, especially in miR-17 family (Figure 1A, Figure 5). Because previous studies believed the well conserved miRNAs across animals rarely secondarily lost [10], [14], [15], [16], we surmised these potential lost haplotypes might be detected in some animal species. For example, network of miR-124 based on 4 haplotypes across 12 animal species showed 2 median vectors (Figure 5), which revealed discontinuous links between these miRNA haplotypes. However, no median vector was found in phylogenetic network of miR-124 family based on 9 haplotypes across 46 animal species (Figure 3). Because more miRNA sequences of miR-124 from more animals were involved, the median vectors had been replaced by some miRNA haplotypes. All of these supported that median vectors should be potential new miRNA sequences that would be found in some specific animal species. Therefore, phylogenetic analysis based on miRNA population across animals would be a method to predict novel miRNA sequences. Here, according to median vectors and links with known miRNAs in phylogenetic network of miR-17 family, we predicted potential miRNA haplotypes in miR-17 family (Table S3).

It is believed that more members exist than known miRNAs in miRBase database (version 13.0, http://microrna.sanger.ac.uk/sequences/). Because less species were studied at miRNA level up to day, many animals are not involved in the curious and interesting miRNA world. As discussed above, we believed that more known miRNA sequences would be detected in other animal species, and more unknown miRNAs or flexible miRNA sequences would be found in species especially at specific times and in specific tissues. Discovering more miRNA data are limited by computational and traditional experimental approaches. Luckily, recently developed “next-generation sequencing” (NGS) technologies, including Illumina sequencing (Solexa sequencing), 454 Life Sciences (Roche), and SOLID™ System (Applied Biosystems), afford an unprecedented opportunity to detect and profile known and novel miRNAs at unprecedented sensitivity. Therefore, it is plausible to detect known miRNA sequences in more species, discover novel miRNAs, and acquire more variations flank mature miRNA sequences. More miRNAs in more species will provide enough data to explore the interesting and complex miRNA world.

### miRNA: An Excellent Phylogenetic Marker

Previous studies showed that evolutionary history of complex miRNA genes could be inferred according to the phylogenetic tree of precursor sequences, such as miR-17 gene family might be experienced through a complex history of duplication and loss of individual members as well as duplication of entire paralogs [20]. Here, by systematic analysis of precursors and mature miRNAs, several evidences showed a complex gene duplication history in the gene family. Firstly, phylogenetic tree of precursors showed all the members of the family were clustered monophyletic groups respectively. The phylogenetic distribution presented that miR-18 gene was the most ancient member (Figure 1B), and the other members might evolve directly or indirectly from the ancestral gene by historical gene duplication event. Secondly, phylogenetic network of miR-17 family based on 31 haplotypes revealed a very complex evolutionary pattern with some median vectors that indicated many lost or potential ancestral miRNA sequences (Figure 1A). According to phylogenetic relationships between haplotypes, evolutionary history and trend can be inferred. Recent miRNA haplotypes were derived directly or indirectly from ancestral haplotypes, lost or potential original miRNA haplotypes by molecular evolution with random genetic drift. Thirdly, haplotype distribution showed miRNA developmental trend across different animal species, especially the evolutionary trend between the members of miR-17 family (Figure 2). Many of the most ancestral haplotypes had lost selection advantage, and more historical or potential ancestral haplotypes had been lost during evolutionary process (Figure 1A, Figure 2). Therefore, the whole miR-17 family with 5 members indicated a complex historical expansion at gene level, such as individual or multiple gene expansion with loss event on the same or different chromosomes. The significant miRNA subdivision with sudden mutation trend would lead to some homologous miRNA genes. Recent miRNA haplotypes derived directly or indirectly from original haplotypes that maybe the most ancestral haplotypes or the lost haplotypes because of random genetic drift and evolutionary fluctuation at DNA level, which would adapt to the specialized miRNA biogenesis machinery [34] and co-evolve with the complex regulatory networks toward a strong evolutionary pressure.

Similarly, for simple miRNA family, miRNAs can be analyzed to infer evolutionary trend and pattern across animal species as population as well as complex miRNA family. For example, haplotype distribution of miR-124 family based on 9 haplotypes indicated hierarchical and discontinuous distribution model across phylogenetic taxa, which might imply miRNA evolutionary trend and correlation with species diversity. The multiple precursors (2∼6) in some chordates might be a response to complex history of gene duplication with less or no variation in miRNA sequences. The network revealed phylogenetic relationships between miRNA haplotypes across animals to dissect evolutionary history and trend. In conclusion, because of highly conserved across the animal kingdom especially the seed sequence (nucleotides 2∼8), miRNA may be an excellent phylogenetic marker as a special population to infer evolutionary pattern, history and trend in the miRNA world.

## Materials and Methods

Both mature and precursor sequences from different animal species of miR-17 (mir-17 gene family, http://microrna.sanger.ac.uk/cgi-bin/sequences/mirna_summary.pl?fam=MIPF0000001) and miR-124 (mir-124 gene family, http://microrna.sanger.ac.uk/cgi-bin/sequences/mirna_summary.pl?fam=MIPF0000021) families were obtained in the miRBase database (version 13.0, http://microrna.sanger.ac.uk/sequences/). Here, we denoted the miRNA precursors by mir-# and the mature miRNAs by miR-# in accordance with the convention in miRBase. To infer possible evolutionary information based on population analysis, we analyzed all mature miRNAs located on the same arms of precursor sequences in different animal species, and simultaneously considered different precursors for the same mature miRNA. Therefore, some miR-17-3p sequences were removed from the paper because of inconsistent with other miR-17 sequences. However, cfa-miR-17 was also removed because it was characterized as cfa-miR-17-3p sequence.

Mature and precursor sequences of miRNAs across animal species were aligned with Clustal X 2.0 [35] respectively by using the multiple alignment. Nucleotide diversity (π) and haplotype diversity (Hd) for the miR-17 and miR-124 families were calculated using DnaSP v. 5.0 [36]. Phylogenetic trees of precursors were reconstructed using the Neighbor-Joining (NJ) method in MEGA 4.0 [37] by 1,000 bootstrap replications. To infer ancestral types of miRNAs, potential types and evolutionary network, the median-joining (MJ, the parameter epsilon was set to 10) phylogenetic network [38] was reconstructed adopting original sequences considered gaps or missing sites with Network 4.5.1.0 (http://www.fluxus-engineering.com). Finally, the maximum parsimony (MP) option [39] was used to identify the unnecessary median vectors and links in the network. Because of these features, the phylogenetic networks of mature miRNA sequences can be reconstructed more precisely. In addition, we also searched for core promoter elements in the consensus region within a 100 kb region upstream of series of miRNA [40].

## Supporting Information

Table S1 Variable sites found in 5 members of miR-17 family across 24 animal species defining 31 haplotypes and their frequencies. Nucleotide positions relative to the beginning of the sequences are indicated by the digits at the top. Sequences identical to H_1 are indicated with dots, and gaps or missing sites are indicated with dashes. * The miRNA haplotype was from miR-106b data.
(0.07 MB DOC)

Table S2 Table S2 Variable sites found in miR-124 family across 46 animal species defining 9 haplotypes and their frequencies. Nucleotide positions relative to the beginning of the sequences are indicated by the digits at the top. Sequences identical to H_1 are indicated with dots, and gaps or missing sites are indicated with dashes.
(0.04 MB DOC)

Table S3 Table S3 Potential novel miRNA sequences in miR-17 family. These sequences were predicted based on phylogenetic relationships among median vectors and known miRNAs in phylogenetic network.
(0.04 MB DOC)
