# Supplementary material for: Haplotype Distribution and Evolutionary Pattern of miR-17 and miR-124 Families Based on Population Analysis
Source: PLoS One. 2009 Nov 23;4(11):e7944. doi: 10.1371/journal.pone.0007944 (PMC2775919; doi:10.1371/journal.pone.0007944)
Supplement: Table S1 — Variable sites found in 5 members of miR-17 family across 24 animal species defining 31 haplotypes and their frequencies. Nucleotide positions relative to the beginning of the sequences are indicated by the digits at the top. Sequences identical to H_1 are indicated with dots, and gaps or missing sites are indicated with dashes. * The miRNA haplotype was from miR-106b data. (0.07 MB DOC) [file pone.0007944.s001.doc]

**Table S1** Variable sites found in 5 members of miR-17 family across 24 animal species defining 31 haplotypes and their frequencies.

| Member | Haplotype (frequency) | 1 | 2 | 4 | 9 | 1 | 1 | 1 | 1 | 1 | 1 | 2 | 2 | 2 | 2 | 2 |
| --- | --- | --- | --- | --- | --- | --- | --- | --- | --- | --- | --- | --- | --- | --- | --- | --- |
|  |  |  |  |  |  | 0 | 1 | 2 | 3 | 7 | 8 | 0 | 1 | 2 | 3 | 4 |
| miR-18 | H_1 (18) | U | A | G | A | U | C | U | A | C | A | A | U | A | - | - |
|  | H_2 (4) | . | . | . | . | . | . | . | . | . | . | . | . | . | G | - |
|  | H_3 (1) | . | . | . | . | . | . | . | U | U | . | U | . | . | - | - |
|  | H_4 (1) | . | . | . | . | . | . | . | . | . | U | U | . | . | G | - |
|  | H_5 (1) | . | . | . | . | . | . | . | . | . | . | U | - | - | - | - |
|  | H_6 (3) | . | . | . | . | . | . | . | . | . | . | U | . | . | - | - |
|  | H_7 (5) | . | . | . | . | . | . | . | . | . | . | U | . | . | G | - |
|  | H_8 (1) | . | . | . | . | . | U | . | . | . | . | . | . | . | - | - |
| miR-17 | H_9 (6) | C | . | A | U | . | A | C | . | . | . | G | . | . | - | - |
|  | H_10 (6) | C | . | A | U | . | A | C | . | . | . | G | . | . | G | - |
|  | H_11 (14) | C | . | A | U | . | A | C | . | . | . | G | . | . | G | U |
| miR-93 | H_26 (11) | - | . | A | U | G | U | . | C | . | . | G | . | . | G | - |
|  | H_27 (1) | C | . | A | U | G | U | . | C | . | . | G | . | . | - | - |
|  | H_28 (4) | C | . | A | U | G | U | . | C | . | . | G | . | . | G | - |
|  | H_29 (1) | - | - | A | U | G | U | . | C | . | . | G | . | . | G | - |
|  | H_30 (1) | A | . | A | U | G | U | . | U | . | . | G | . | . | - | - |
| miR-106 | H_16 (1) | C | . | A | U | A | A | C | . | . | . | G | . | . | G | - |
|  | H_17 (1) | - | . | A | U | . | A | C | . | . | . | G | . | . | G | - |
|  | H_18 (9) | A | . | A | U | . | A | C | . | . | . | G | . | . | G | C |
|  | H_19 (1) | A | . | A | U | . | A | C | . | . | . | G | . | - | - | - |
|  | H_20 (2) | A | . | A | U | . | A | C | . | . | . | G | . | . | - | - |
|  | H_21 (1) | A | . | A | U | . | A | C | . | . | . | G | . | . | G | - |
|  | H_31 (1) | A | . | A | U | . | A | . | . | . | . | G | . | . | G | A |
|  | H_22 (14)^*^ | . | . | A | U | G | A | C | . | . | . | . | . | - | - | - |
| miR-20 | H_12 (8) | C | . | A | U | C | A | . | . | . | . | G | . | . | G | - |
|  | H_13 (1) | C | . | A | U | C | A | . | . | . | . | G | . | - | - | - |
|  | H_14 (2) | C | . | A | U | C | A | C | . | . | . | G | . | . | - | - |
|  | H_15 (1) | C | . | A | U | C | A | C | . | . | . | G | . | . | G | - |
|  | H_23 (2) | . | . | A | U | . | A | . | . | . | . | G | - | - | - | - |
|  | H_24 (11) | . | . | A | U | . | A | . | . | . | . | G | . | . | - | - |
|  | H_25 (11) | . | . | A | U | . | A | . | . | . | . | G | . | . | G | - |

Nucleotide positions relative to the beginning of the sequences are indicated by the digits at the top. Sequences identical to H_1 are indicated with dots, and gaps or missing sites are indicated with dashes. ^*^ The miRNA haplotype was from miR-106b data.
