# Supplementary material for: Haplotype Distribution and Evolutionary Pattern of miR-17 and miR-124 Families Based on Population Analysis
Source: PLoS One. 2009 Nov 23;4(11):e7944. doi: 10.1371/journal.pone.0007944 (PMC2775919; doi:10.1371/journal.pone.0007944)
Supplement: Table S2 — Variable sites found in miR-124 family across 46 animal species defining 9 haplotypes and their frequencies. Nucleotide positions relative to the beginning of the sequences are indicated by the digits at the top. Sequences identical to H_1 are indicated with dots, and gaps or missing sites are indicated with dashes. (0.04 MB DOC) [file pone.0007944.s002.doc]

**Table S2** Variable sites found in miR-124 family across 46 animal species defining 9 haplotypes and their frequencies.

| Haplotype (frequency) | 1 | 1 | 1 | 2 | 2 | 2 | 2 |
| --- | --- | --- | --- | --- | --- | --- | --- |
|  |  | 0 | 2 | 1 | 2 | 3 | 4 |
| H_1 (2) | U | G | A | C | A | - | - |
| H_2 (14) | . | . | G | . | . | - | - |
| H_3 (1) | . | . | G | . | . | A | - |
| H_4 (9) | - | . | G | . | - | - | - |
| H_5 (11) | - | . | G | . | . | - | - |
| H_6 (18) | - | . | G | . | . | A | - |
| H_7 (18) | - | . | G | . | . | A | G |
| H_8 (1) | - | . | G | U | . | A | - |
| H_9 (1) | - | U | G | U | . | A | - |

Nucleotide positions relative to the beginning of the sequences are indicated by the digits at the top. Sequences identical to H_1 are indicated with dots, and gaps or missing sites are indicated with dashes.
