# Supplementary material for: Haplotype Distribution and Evolutionary Pattern of miR-17 and miR-124 Families Based on Population Analysis
Source: PLoS One. 2009 Nov 23;4(11):e7944. doi: 10.1371/journal.pone.0007944 (PMC2775919; doi:10.1371/journal.pone.0007944)
Supplement: Table S3 — Potential novel miRNA sequences in miR-17 family. These sequences were predicted based on phylogenetic relationships among median vectors and known miRNAs in phylogenetic network. (0.04 MB DOC) [file pone.0007944.s003.doc]

**Table S3** Potential novel miRNA sequences in miR-17 family. These sequences were predicted based on phylogenetic relationships among median vectors and known miRNAs in phylogenetic network.

| Median vector | Sequence (5'-3') |
| --- | --- |
| mv4 | CAAAGUGCUCAUAGUGCAGGUA |
| mv11 | AAAAGUGCUUACAGUGCAGGUAGA |
| mv12 | AAAAGUGCUUAUAGUGCAGGUAG |
| mv16 | CAAAGUGCUCACAGUGCAGGU |
| mv17 | UAAAGUGCUUAUAGUGCAGGU |
| mv21 | AAAAGUGCUGUUCGUGCAGGUA |
| mv30 | CAAAGUGCUGAUAGUGCAGGUAG |
| mv38 | UAAAGUGCUUACAGUGCAGGU |
| mv42 | CAAAGUGCUGAUAGUGCAGGUA |
| mv43 | AAAAGUGCUGUUAGUGCAGGUA |
| mv53 | CAAAGUGCUGAUAGUGCAGGU |
| mv56 | UAAAGUGCUGACAGUGCAGGU |
| mv58 | UAAAGUGCUCACAGUGCAGGU |
| mv63 | CAAAGUGCUGUUAGUGCAGGUA |
| mv64 | CAAAGUGCUGUUUGUGCAGGUA |
| mv65 | CAAAGUGCUGUUAGUGCAGGUAG |
| mv79 | UAAAGUGCUCAUAGUGCAGGU |
| mv81 | UAAAGUGCUGAUAGUGCAGGU |
| mv111 | UAAAGUGCUGUUAGUGCAGGUA |
| mv119 | UAAAGUGCUGUUCGUGCAGGUA |
| mv122 | UAAAGUGCUGUUUGUGCAGGUA |
| mv125 | UAAGGUGCAUCUAGUGCAGGUA |
| mv126 | UAAGGUGCAUAUAGUGCAGAUA |
| mv129 | UAAAGUGCUUUUAGUGCAGGUA |
| mv130 | UAAGGUGCAUCUAGUGCAGGUAG |
| mv133 | UAAAGUGCUUCUAGUGCAGGUAG |
| mv136 | UAAGGUGCAUUUAGUGCAGGUA |
| mv143 | UAAGGUGCAUAUAGUGCAGGUA |
